# Distinct early cortical thickness patterns in heterozygous APOE3‐Christchurch carriers and age‐matched controls

**DOI:** 10.1002/alz70856_097335

**Published:** 2025-12-24

**Authors:** Vincent Malotaux, David Fernando Aguillón Niño, Isabela Gonzalez, Lusiana Martinez, Sergio Alvarez, Justin S Sanchez, Catarina Tristão‐Pereira, Bing He, Averi Giudicessi, Diego Sepulveda‐Falla, Daniel Vasquez, Yamile Bocanegra, Lucia Madrigal, Diana Alzate, Claudia Muñoz, Clara Vila‐Castelar, Liliana A Ramirez Gomez, Joseph F Arboleda‐Velasquez, Ibai Diez, Yakeel T. Quiroz

**Affiliations:** ^1^ Massachusetts General Hospital, Harvard Medical School, Boston, MA, USA; ^2^ Department of Psychological & Brain Sciences, Boston University, Boston, MA, USA; ^3^ Grupo de Neurociencias de Antioquia, Universidad de Antioquia, Medellín, Colombia; ^4^ Hospital Pablo Tobón Uribe, Medellín, Antioquia, Colombia; ^5^ Washington University School of Medicine, St. Louis, MO, USA; ^6^ Boston University, Boston, MA, USA; ^7^ Institute of Neuropathology, University Medical Center Hamburg‐Eppendorf, Hamburg, Germany; ^8^ Grupo de Neurociencias de Antioquia, Universidad de Antioquia, Medellín, Antioquia, Colombia; ^9^ Grupo de Neurociencias de Antioquia, Facultad de Medicina, Universidad de Antioquia, Medellín, Colombia; ^10^ Schepens Eye Research Institute of Mass Eye and Ear and Department of Ophthalmology, Harvard Medical School, Boston, MA, USA; ^11^ Grupo de Neurociencias de Antioquia, Facultad de Medicina, Universidad de Antioquia, Medellín, Antioquia, Colombia

## Abstract

**Background:**

The APOE3‐Christchurch (APOE3^Ch^) variant has been linked to protection against Alzheimer's disease (AD), even in the presence of substantial genetic and pathological risk factors. Although the exact mechanisms underlying this protective effect are not fully understood, structural neuroimaging could shed light on early brain changes associated with AD resistance. In this study, we analyzed cortical thickness (CT) in middle‐aged heterozygous APOE3^Ch^ carriers and compared them to age‐matched non‐carrier family members.

**Methods:**

We included 52 non‐demented individuals with Mini‐Mental State Exam (MMSE) scores of ≥24/30, comprising 15 heterozygous APOE3^Ch^ carriers (aged 40.2±12.4 years, 53% female) and 37 non‐carriers (aged 40.7±5.6 years, 54% female). All participants underwent structural MRI at the Pablo Tobon Uribe Hospital in Colombia. Cortical differences between APOE3^Ch^ carriers and non‐carriers were assessed using voxel‐based morphometry analyses, adjusted for age. Additionally, CT across 68 regions of interest (ROI), defined by the Desikan‐Killiany atlas in FreeSurfer, was extracted and analyzed. Comparisons included ROIs and an AD‐related meta‐ROI (Metha et al. 2024), using multiple linear regression adjusted for age. Clinical measures included MMSE and Functional Assessment Staging Tool (FAST) scores.

**Results:**

Heterozygous APOE3^Ch^ carriers had similar MMSE and FAST scores to non‐carriers (mean MMSE: APOE3^Ch^ carriers=28.40±0.99, non‐carriers=28.76±1.14, *p=*.27; mean FAST: APOE3^Ch^ carriers=1.4±0.63, non‐carriers=1.05±0.23, *p* = .06). Both groups had similar sex distribution (χ^2^=0.002, *p=*.96), though APOE3^Ch^ carriers reported fewer years of formal education compared to non‐carriers (mean education: APOE3^Ch^ carriers=8.33±3.99 years, non‐carriers=12.24±3.66 years, *p=*.003). Whole‐brain voxel‐wise and ROI analyses revealed increased CT in frontal and parietal regions of the APOE3^Ch^ carriers, alongside reduced thickness in occipital and temporal regions. Cortical thickness within the AD‐related meta‐ROI was comparable between APOE3^Ch^ carriers and non‐carriers (mean CT: APOE3^Ch^ carriers=2.50±0.07mm, non‐carriers=2.47±0.10mm, *p* = .23).

**Conclusion:**

Heterozygous APOE3^Ch^ carriers demonstrate distinct early CT patterns, characterized by increased thickness in fronto‐parietal regions. These areas have been associated with enhanced cognitive reserve and may play a role in delaying the onset of AD. These findings suggest that structural differences in specific brain regions may contribute to the protective effects observed in APOE3^Ch^ carriers. Future research incorporating multimodal brain imaging could help us uncover the mechanisms driving AD resistance in these individuals.

